# Using mosquito and arbovirus data to computationally predict West Nile virus in unsampled areas of the Northeast United States

**DOI:** 10.1093/pnasnexus/pgaf227

**Published:** 2025-08-19

**Authors:** Joseph R McMillan, James Sun, Luis Fernando Chaves, Philip M Armstrong

**Affiliations:** Department of Biological Sciences, Texas Tech University, 2901 Main St., Lubbock, TX 79409, USA; Clark Scholars Program, Department of Biological Sciences, Texas Tech University, 2901 Main St., Lubbock, TX 79409, USA; Clark Scholars Program, Department of Biological Sciences, Texas Tech University, 2901 Main St., Lubbock, TX 79409, USA; Department of Environmental and Occupational Health, School of Public Health and Department of Geography, Indiana University, 1025 E. Seventh Street, Suite 111, Bloomington, IN 47405, USA; Department of Entomology, The Connecticut Agricultural Experiment Station, 123 Huntington St., New Haven, CT 06511, USA

**Keywords:** machine learning, hierarchical modeling, West Nile virus, *Culex pipiens*, risk mapping

## Abstract

Predicting and projecting risk of West Nile virus (WNV) to humans in areas without mosquito surveillance data is a key limitation of many WNV surveillance programs. To better inform risk of WNV, we analyzed 20 years (2001–2020) of point-level mosquito surveillance data from Connecticut (CT), United States, using machine learning methods to determine the most informative weather variables and land cover classes associated with monthly *Culex pipiens* collections and WNV detections in mosquitoes. All training models were assessed based on explained deviance, root mean square error, and parsimony of included variables then optimized using a backward selection process. We used these training models to create a predictive mapping framework that could spatially extrapolate the monthly risk of WNV activity in mosquitoes across the entirety of the Northeast United States (CT, Maine, Massachusetts, New Hampshire, New Jersey, New York, Rhode Island, and Vermont) at a 4 × 4 km resolution. We then validated WNV detection probabilities against observed human cases at the town level in CT and the county level for northeastern states using generalized linear (mixed effects) models. Our predicted town- and county-level WNV detection probabilities in mosquitoes were significantly associated with the odds of a human case occurring within the town and/or county. This methodology increases the utility of point-source mosquito surveillance data by creating a flexible workflow for predicting risk of WNV to humans across the Northeast United States using easily accessible online data sources.

Significance statement West Nile virus (WNV) transmission risk is often communicated through risk maps to show where infected humans and/or mosquitoes have been observed. Such spatial displays might under- or over-estimate risk based on the level of surveillance within an administrative unit. Here, we propose a computational methodology that mines a rich and comprehensive mosquito surveillance database to predict the probability of detecting WNV throughout the Northeast United States. We validated our methods using observed data not used in model development and found that our models can accurately predict WNV detection probabilities throughout this region. Our methods increase the value of information obtained from point-source surveillance by predicting WNV risk to the public in regions that are not monitored by mosquito traps.

## Introduction

Vector-borne disease (VBD) transmission is an inherently focal process that is influenced by a myriad of biological, climatic, environmental, and sociodemographic factors (1). Despite the localized and ultrafine scale nature of VBD transmission, patterns can emerge through the aggregation of data at increasing spatial and temporal levels (2, 3). These patterns can identify signals of public health risk which can be predicted (i.e. estimate future risk or risk in unsampled areas) across various time frames and political units. Such outcomes are central to public health messaging for VBDs. The diseases, datasets, and methods used to understand VBD risks to humans vary widely, though all methods aggregate and extrapolate information from sampled to un(der)-sampled areas. The West Nile virus (WNV) transmission system in the United States is an excellent case study for understanding and predicting spatiotemporal risk. Since WNV was first detected in New York, NY in 1999, over 50,000 clinical cases have been reported in the United States (4, 5). WNV is maintained by *Culex* spp. mosquitoes and birds in a sylvatic cycle, and humans are considered dead end hosts (i.e. viremia is too low to infect a blood feeding mosquito). Human exposure to WNV occurs when wildlife transmission cycles spill over into human habitats and populations. Severe clinical infections of WNV are rare, yet it is estimated that only 1% of all human infections are reported (4 , 6). Due to this large though uncertain public health burden of disease, there have been tremendous efforts to predict and project the spatiotemporal dynamics of WNV epidemics in the United States and abroad.

Multiple research objectives have driven research into predicting WNV risk to humans, including determining climate factors that increase seasonal risk (7, 8), exploring range expansions under climate change scenarios (9), forecasting incidence at a county level in real-time (10, 11), and developing methodologies that treat WNV clinical incidence as a probabilistic rather than deterministic process (12, 13). In the United States, the unit of exploration in models of human WNV incidence is typically counties, as this is the level at which human cases are reported to the Centers for Disease Control and Prevention. Models of WNV incidence in humans have also proven successful at predicting disease incidence without including direct measures of WNV activity in the sylvatic cycle (e.g. infection rates in mosquitoes or birds); however, the available localized models of human WNV incidence do show that human cases typically peak at a lag from peak infections in mosquitoes (14). Predictive studies of WNV activity in mosquitoes focus on examining the factors that influence mosquito infection rates at specific sites in an area typically no larger than a city or county (15–18) (with some exceptions), and these studies have shown that hotter, drier, and more urban spaces for *Culex pipiens* Linnaeus*/Culex quinquefasciatus* Say and rural spaces for *Culex tarsalis* Coquillett during the summer months strongly predict the intensity of WNV mosquito infections (19–23). Together, the typical public health risk message from these studies is that broad climatic patterns (either mild winters, wet springs, or hot and dry summers) and certain classifications of land use (rural vs. urban) are associated with increased risk of WNV activity in mosquitoes and subsequent exposure in humans. These messages, however, do little to pinpoint spatial risk based on reported WNV activity from surveillance systems.

The actual practice of communicating risk of WNV to the public in real-time relies on operating active and/or passive WNV surveillance system(s). Passive WNV systems consist of reporting human/equine infections and/or dead birds as they are detected by medical professionals and veterinarians. Active systems are more flexible in terms of where surveillance can occur, and these systems typically consist of trapping adult mosquitoes in various high-risk spaces and testing subsets of mosquitoes for WNV. The location of positive traps or positive jurisdictions may then be reported in maps used to warn the public that WNV is active in a sampled area. Although risk may be assessed at the level of a trap, the general WNV risk communication issue becomes: how do active surveillance programs communicate the risk of WNV to residents in jurisdictions that do not contain an active surveillance trap (e.g. traps collecting mosquitoes in the field)? This is a common communication issue faced by the Connecticut Agricultural Experiment Station (CAES, New Haven, CT, USA) which operates a statewide mosquito and arbovirus surveillance system for multiple arboviruses of public health concern.

CAES established their mosquito and arbovirus surveillance systems for eastern equine encephalitis virus (EEEV) in 1997; the system now currently monitors mosquito populations and arbovirus activity at 108 sites in Connecticut (CT) (24, 25): major expansions occurred in 2001 (due to WNV) and 2019 (due to EEEV). To improve the capabilities of CAES and other WNV surveillance systems in the Northeast United States to communicate the risk of WNV in mosquitoes to all residents within an administrative unit, we analyzed longitudinal mosquito, arbovirus, and human case data from the state of CT, United States using a series of hierarchical boosted regression tree models. We trained a series of nested hierarchical models to predict WNV detection probabilities in *Cx. pipiens* mosquitoes using a subset of the CAES mosquito surveillance dataset in combination with multiple weather, land cover, and demographic variables. We then validated these risk models by examining the relationships between predicted town-level (CT only) and county-level (Northeast states—New Jersey, New York, CT, Rhode Island, Massachusetts, Vermont, New Hampshire, and Maine) WNV detection probabilities and observed WNV infections in humans. From these results, we can create monthly, statewide risk maps of WNV detection probabilities in mosquitoes which can be used by local public health offices throughout the Northeast United States to assess WNV spatial risk based on current and projected climate data in areas with and without mosquito surveillance.

## Methods

All analyses took place in R V4.2.2 (26) using a variety of packages listed in each subsection. Any data extracted from a raster image was extracted and averaged across a 5 km buffer of each surveillance site using the extract function in the “raster” package. We chose a 5 km buffer surrounding each trap to estimate these relationships since prior analyses with the CAES surveillance data set identified spatial patterns of synchronous WNV detection in mosquitoes up to 5 km (27). All spatial data used, or was reprojected to, the WGS84 coordinate projection system (CRS). Conversions to this CRS, when needed, were performed using the corresponding functions available in the “raster” and “sf” packages (28, 29). All displayed maps were created using a combination of the “maps”, “sf”, and “ggplot2” packages (29, 30). A graphical abstract of our workflow is available in Fig. S1.

### Training data

To develop our modeling framework, we aggregated multiple data sources from 2001 to 2020 including CAES mosquito and arbovirus data, monthly weather data (i.e. temperature, precipitation, and drought conditions), static land cover data, and static human population density data (which was used as an additional proxy for urbanization). Detailed descriptions of data acquisition, geoprocessing, and extraction to site level can be found in Supporting Information: Methods. General descriptions are included below.

### Mosquito and arbovirus surveillance data

Mosquito and WNV data were obtained using hay-lactalbumin infusion-baited gravid traps at 87 surveillance sites in CT from 2001 to 2020. These traps are the most efficient surveillance tool for detecting WNV-infected *Cx. pipiens* mosquitoes (31), which is the primary enzootic and epidemic vector of WNV in the US northeast (32, 33). Briefly, sites were sampled by operating traps overnight on a 10-day rotation each summer from June through October, and all female mosquitoes were identified to species using a dichotomous key (34) and tested for nine arboviruses using viral cell culture and RT-PCR techniques (35). Collection sites were sampled more frequently (1–2 times weekly) for the remainder of a season if mosquitoes tested positive for an arbovirus of primary public health concern (WNV or EEEV); gravid traps were employed in all supplemental sampling events regardless of if the arbovirus under investigation is WNV or EEEV. For our modeling purposes, we chose to aggregate mosquito collections and WNV detections to the scale of a month corrected for the number of sampling events that took place at a site during a given month. Our calibration models of WNV detection modeled detection as 1—at least one trap night per month contained WNV positive mosquitoes and 0—no trap nights contained WNV positive mosquitoes per month. This approach generates a model that is trained to recognize patterns associated with (in)consistent WNV detection events in space and time. Given our ultimate objective is to map spatial risk of WNV activity across CT, this approach is appropriate. See Supporting Information: Methods for greater detail on variable choice.

### Weather data

Monthly average temperature and precipitation records from 2001 to 2020 as well as monthly climate 30-year normals were obtained from PRISM (36) by extracting data to the specific surveillance site coordinates using the Data Explorer tool (https://prism.oregonstate.edu/explorer/). PRISM uses a default 4 km grid system, meaning weather values are assigned to points based on the grid in which sites are located. In all training models, we included the current month and a 1-month lag in our weather variables.

### Drought conditions

Monthly drought conditions, defined using the Palmer Drought Severity Index (PDSI), were obtained from the National Oceanic and Atmospheric Administration. PDSI is a composite score of dryness calculated as a cumulative departure of surface water balance (37) and is reported on a standardized scale of −10 (extremely dry) to +10 (extremely wet). NOAA reports PDSI metrics at multiple geopolitical and environmental units. For the purposes of our modeling work, we extracted PDSI values to the point level based on NOAA climatic zones. In all training models, we included the current month and a 1-month lag in PDSI.

### Landcover data

Landcover data were obtained from the Multi-Resolution Land Characteristics Consortium (https://www.mrlc.gov/data?f[0]=project_tax_term_term_parents_tax_term_name:Annual NLCD). Briefly, this site hosts annual data on 16 land cover class estimates across the United States at annual intervals. At the time of analysis, not all annual years were readily available from MRLC; our training models utilized data estimated from 2001, 2004, 2006, 2008, 2011, 2013, 2016, and 2019. We modeled land cover as the percentage land cover of each type within 5 km of a surveillance site.

### Human population density data

Because numerous field studies in the eastern and Midwestern United States identify urban habitats as important metrics of WNV risk in mosquitoes and humans, we included human population density data as an additional indicator of urbanization in our training models. While human population density alone may not fully capture some of the nuances of urban vs. rural infrastructure variability (38, 39), it is associated with habitats essential for *Cx. pipiens* population dynamics (such as catch basin density and/or percent impervious surface (40)). In this investigation, it was assumed that these metrics are likely correlated, and population density sufficiently captures metrics of US urban infrastructure.

We downloaded the gridded global population density raster image for 2000, 2005, 2010, 2015, and 2020 from Columbia University's Socioeconomic Data and Applications Center (https://sedac.ciesin.columbia.edu/data/set/gpw-v4-population-density-adjusted-to-2015-unwpp-country-totals-rev11/data-download), then extracted the average population density estimates within 5 km of each surveillance site.

### Model development

All training models utilized monthly variables. Response variables were either *Cx. pipiens* monthly trap night collections or WNV detection (1—one or more positive trap nights in a month, 0—no positive trap nights in a month). The full list of predictor variables is available in Table S1.

We used three types of modeling approaches to calibrate models of *Cx. pipiens* monthly trap night collections and WNV monthly detections. We used gradient boosting machines, specifically boosted regression trees (BRT), in the “dismo” and “gbm” R packages (41) as our primary modeling approach. Full models of *Cx. pipiens* collections or WNV detection included all 15 land cover classes, a subset of monthly and 1-month lag weather variables, monthly and 1-month lag PDSI, and population density within 5 km of a surveillance site; full models of WNV detection (1—one or more positive trap nights in a month, 0—no positive trap nights in a month) included the same variables with the addition of the observed *Cx. pipiens* monthly trap night collection. All candidate BRT models were simplified through a backward elimination approach with explained deviance calculated with each variable removal. We then compared simplified candidate BRT models to an all pairwise interaction general linear model (GLM) and to a random forest (RF) regression (*Cx. pipiens* collections) or classification (WNV detections) using the simplified BRT's final terms; GLMs and RFs were further simplified using a backward selection procedure based on AIC (GLMs) or variable importance (RFs). The best performing models were assessed from the vantage points of model deviance, root mean square error (RMSE, assessed using the test data), and parsimony (i.e. fewest variables). Final models utilized for validation were chosen based on lowest RMSE of the simplified BRT and GLMs.

### Model validation

We performed two different model validation procedures. See Supporting Information: Methods for detailed descriptions on how training data was acquired and transformed for each validation approach. First, we compared the predicted probability of WNV monthly detections in mosquitoes from our final candidate models to the observed presence/absence of WNV detections in humans in CT 2001–2022. We used binomial-error generalized mixed effects models with observed WNV detections in humans (modeled as detected 1, not detected 0 in any month) at the town level as a response variable, the average annual predicted WNV mosquito detection probability as a fixed effect, the town's human population density (total population divided by town area, log transformed) as an intercept offset, and town name and year of collection as a random effects (to control for repeated observations (42)). We chose human case occurrence rather than absolute number of cases since there were rarely more than one human case for any given year, town, and month combination.

Our second validation approach was to compare predicted WNV detection probabilities in mosquitoes to observed detections of human cases throughout Northeast counties 2021–2022. Human data at a national level is reported on at the scale of a US county on an annual basis, and we used a generalized linear hurdle model framework to test the associations between our predicted county-level WNV infection probabilities in mosquitoes and observed detections of human cases. These models exclusively accounted for human population density as a predictor of a human case at the county level because many counties in the northeast far exceed the observed population density modeled in CT. After accounting for these relationships, the hurdle model then analyzed the number of human cases in a county as a function of predicted probabilities of detecting WNV in *Cx. pipiens* mosquitoes.

## Results

### Summary data

**Fig. 1.**
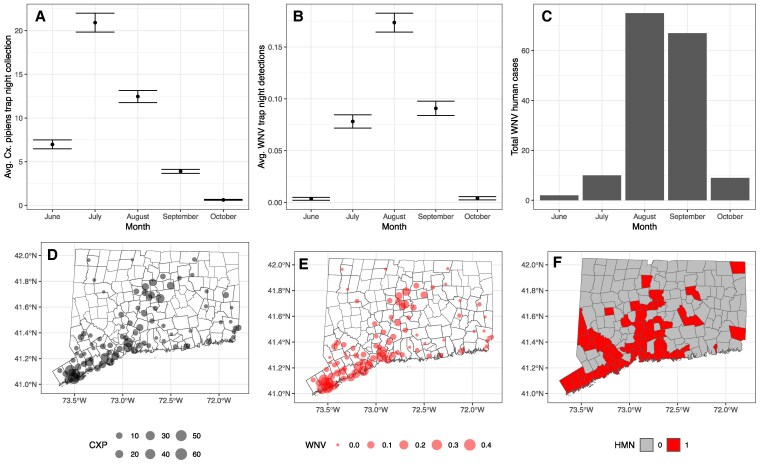
Summary data: A) Average (±SE) monthly *Cx. pipiens* trap night collections, B) WNV monthly trap-night positivity rates, C) total human cases in CT, United States by month of the year from 2001 to 2020. Maps containing points display the location of 87 surveillance traps: D) the average trap night collection of *Cx. pipiens* mosquitoes, E) the average monthly detection of WNV, and F) the human incidence maps display the presence/absence of any human case across all years and months of sampling at the level of a town. Shapefiles for CT boundaries and jurisdictional units were obtained from CT DEEP GIS Open data (https://deepmaps.ct.gov/).

From 2001 to 2020, CAES performed 1,501 trap night collections using gravid traps which resulted in captured and tested 261,092 *Cx. pipiens* females tested for WNV in 14,871 total mosquito pools of which 1,417 were WNV positive. During that time, 163 human cases were detected in CT. An additional 203 WNV positive *Cx. pipiens* pools from gravid traps and 13 human cases were detected between 2021 and 2022. *Culex pipiens* collections peaked in July while peak prevalence of WNV in mosquitoes occurred in August as did the peak in reported human cases (Fig. 1A–C). WNV detections in mosquitoes declined sharply from August to October while human case reports were similar between August and September (Fig. 1A–C). All three variables—*Cx. pipens* collections, WNV detection in mosquitoes, and occurrence of human cases of WNV—were higher in the southwestern and central portions of CT (Fig. 1D–F).

### Model development

Variable inclusion for all training models of monthly *Cx. pipiens* trap night collections and WNV detection probabilities is listed in Table S2. Model performance results are available as Supporting Information.

### *Culex pipiens* monthly trap night collections

After giving equal weight to the number of additive and interactive variables, percent explained deviance, percent explained residual deviance, and RMSE (Tables S3–S5), the best performing boosted regression tree (BRT) model for *Cx. pipiens* monthly trap night collections included six terms: current month PDSI, human population density, percent wetland forest within 5 km of a trap site, prior month PDSI, current month difference from normal precipitation, and percent emergent wetland within 5 km of a trap site (Table S2, Fig. S2). While BRT predictions are inherently nonlinear, general variable relationships with *Cx. pipiens* trap night collection for the top five most important variables were as follows: drought—negative, population density—positive, wetland forest—u-shaped, prior month PDSI—positive, precipitation difference from normal—positive (Fig. S3). The best fitting BRT model was further reduced to five variables in a RF regression (wetland forest was dropped) (Table S2, Fig. S2). The best fitting reduced GLM contained 13 additive terms (Table S3, Fig. S2): additional land cover variables beyond variables listed above included coniferous forest, pasture, developed classes 2 and 3, and barren; current month weather variables were averages while prior month variables were difference from normal. Boosted regression tree models tended to outperform other model forms in terms of explained deviance while GLMs tended to outperform other model forms in terms of RMSE (Tables S3–S5). After inspecting residuals and prediction plots at the scale of CT, it was determined that GLMs were over-parameterized and would predict monthly *Cx. pipiens* trap night collections in the millions (see Vint and Vimp scales in Fig. S2 for the final GLM of *Cx. pipiens* models); therefore, GLMs of *Cx. pipiens* collections were excluded from further consideration.

### Monthly WNV detection probability

The best performing BRT model for WNV monthly detections (Fig. S1, Table S6) included 12 terms: *Cx. pipiens* monthly trap night collections, prior month average temperature, human population density, prior month PDSI, prior month average precipitation, current month temperature and precipitation difference from normal, and percent develop class 4, wetland forest, grass, and scrub within 5 km of a trap site (Fig. S2). General variable relationships with monthly WNV detection probabilities for the top five variables were as follows (Fig. S4): *Cx. pipiens* trap night collection—positive, prior month temperature—positive, population density—positive, prior month PDSI—humped shaped, and prior month average precipitation—u-shaped. As with *Cx. pipiens* models, BRT model forms outperformed GLMs and RFs by explained deviance, though RMSE values were similar between BRTs and GLMs (Tables S6–S8). RF classification models had extremely high sensitivity as well as extremely low specificity and thus did not reliably identify spaces as WNV positive (Table S7). Partial dependence plots revealed similar variable functional relationships between variable and response terms (Figs. S4–S6) across model types; however, like GLMs of *Cx. pipiens* collections, GLMs of WNV detections tended to be overparameterize and overfit the data. Because of the determined limitations of RF and GLM iterations of our *Cx. pipiens* and WNV models, we moved forward only using BRT versions.

### Model validation

**Fig. 2.**
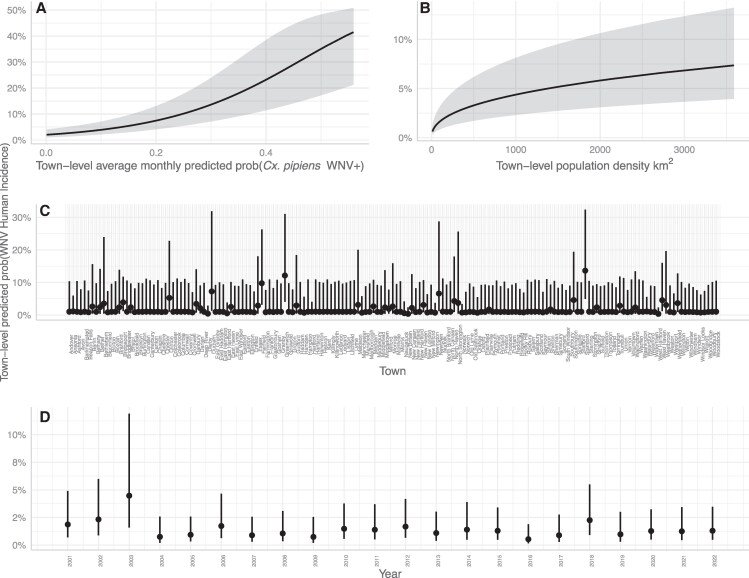
Model validation results for CT 2001–2022: A) The predicted probability of observing a WNV human case in a CT town based on town-level WNV detection probabilities in *Cx. pipiens* mosquitoes. B) The predicted relationship between town-level human population density and the predicted probability of observing a WNV human case. C) The predicted probability of detecting a human case in the 169 towns in CT. D) The predicted probability of observing a human case in each year of sampling. Predictions were generated using a generalized linear model with town-population size as an intercept offset, the average monthly town-level WNV detection probabilities in *Cx. pipiens* mosquitoes as a fixed effect, and town and year as random effects. Lines with shaded regions represent the average relationship and its 95% CI; points with lines represent the average relationship and its 95% CI.

Using only the best fitting BRTs of *Cx. pipiens* trap night collections and WNV detections, validation approaches confirmed the utility of predicted monthly WNV detection probabilities in mosquitoes for predicting spatial risk of WNV activity within CT as well as across the northeast United States. For validation of models within a CT town from 2001 to 2022, average annual WNV detection probabilities in mosquitoes were positively associated with an increased probability of a human case occurring within a town, with the probability of a human case being detected increasing from 0 to ∼40% as the predicted probability of WNV being detected in mosquitoes increased from 0 to ∼55% (Fig. 2); this effect was much greater than that of human population size which was only predicted to increase the probability of human detection from 0 to ∼7%. There was some variation attributable to year and town (which were modeled as random effects) and some towns and years displayed significant differences from the model's intercept; however, all CIs overlapped between random effect terms (Fig. 2).

**Fig. 3.**
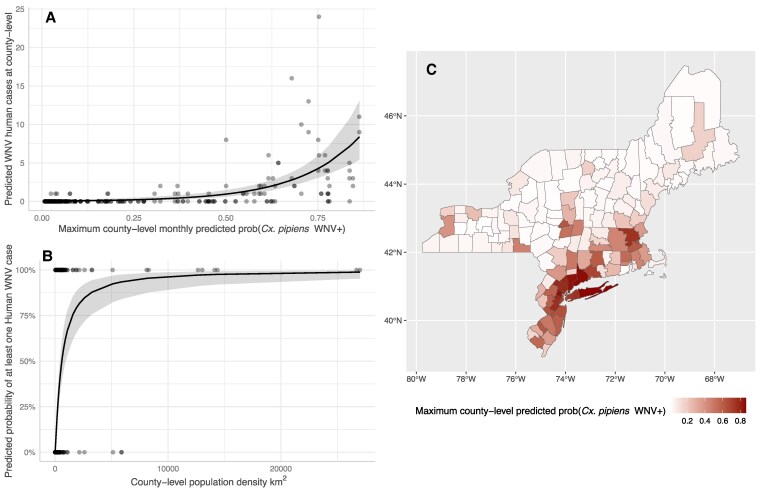
Model validation results for the Northeast United States 2021–2022: A) Predicted WNV human cases in a northeast county 2021–2022 based on the maximum predicted county-level WNV detection probabilities in *Cx. pipiens* mosquitoes. B) The predicted relationship between county-level human population density and the predicted probability of observing a WNV human case. C) Map of maximum predicted county-level WNV detection probabilities in *Cx. pipiens* mosquitoes. Predictions were generated using a generalized linear hurdle model which first fits a relationship between presence/absence of a human case predicted by county-population density and then fits a relationship between the number of human cases (>0) and the maximum predicted WNV detection probabilities in *Cx. pipiens* mosquitoes within a county. Lines with shaded regions represent the average relationship and its 95% CI; black dots represent observed data. Predicted relationships are equivocal whether human population density is modeled as an intercept offset or as a predictor of presence/absence of a human case. The map is provided to aid viewers in the spatial distribution of WNV risk predicted in the northeast during the investigated time period. Shapefiles for NE boundaries and jurisdictional units were obtained from CT DEEP GIS Open data (https://deepmaps.ct.gov/).

For validation of models within the northeast from 2021 to 2022, the presence/absence of a human case is strongly tied to the number of humans living within a county (Fig. 3), with the probability of a human case being detected quickly increasing from 0 to ∼99% as population density increases. After accounting for this relationship, we found a significant and positive association with the predicted probability of WNV being detected in mosquitoes and the number of human cases reported in a county (Fig. 3). A map of the seasonal maximum predicted probability of WNV infections in *Cx. pipiens* mosquitos is also provided in Fig. 3, which shows the highest probabilities of WNV activity are located along southern and eastern edge of the Northeast, reflecting the high prevalence of urbanized spaces in these areas (43).

### Model application

**Fig. 4.**
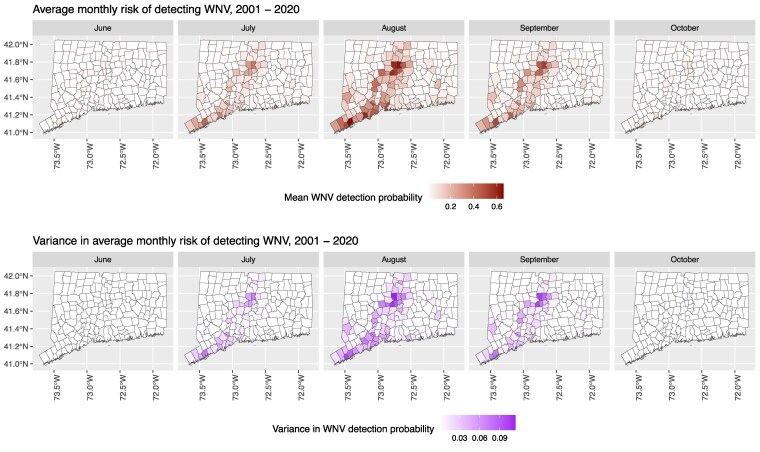
CT-based WNV risk maps: Monthly risk maps of predicted WNV detection probabilities in *Cx. pipiens* mosquitoes in CT towns 2001–2020. Top row is the average monthly value across years; the bottom row is the variance in predicted values across years. Shapefiles for CT boundaries and jurisdictional units were obtained from CT DEEP GIS Open data (https://deepmaps.ct.gov/).

Static average seasonal risk maps of WNV activity in *Cx. pipiens* mosquitoes are displayed in Fig. 4. Our model accurately captures the highest risk of WNV in the summer months (July–September) in the most urban of spaces (e.g. coastal regions in the southwest portion and central CT). Variance in average monthly predictions was also highest during these time periods and in these regions, reflecting our model's ability to capture variation in weather impacts on WNV transmission between seasons (see Fig. S7 for a mean by variance plot). We provide two further examples of the model's utility to communicate risk. Figure S8 displays seasonal predicted risk in 2004 (which was a low prevalence year for WNV with only nine WNV positive pools from gravid traps) and in 2018 (which recorded the highest number and prevalence of seasonal WNV positive pools in CAES history: 255 positive pools from gravid traps). The spatial expansion and magnitude of risk that defined the 2018 transmission season is captured by the model predicting increased risk of WNV in the areas surrounding CT's urban cores as well very high detection probabilities within the cores; expansion of risk into eastern CT is also captured by the model, especially in the month of August. Figure S9 displays predicted risk of WNV based on user-specified null weather values (for variables that were either indexed, such as PDSI, or represented a difference from normal, we set all null values to 0. For variables that were an observed monthly value, we set null values to the specific month's normal value from the historical record). These figures provide baseline estimates of risk that can be easily altered to re-project risk based on current and future weather conditions as made available by different weather prediction systems.

## Discussion

To enhance the utility of a current public health surveillance system to project risk of WNV within the entire political boundary of a US state and region, we developed a series of nested models that first predict the monthly average trap night collection of *Cx. pipiens* mosquitoes; these monthly trap night predictions were then used to predict the risk of detecting WNV in captured mosquitoes. Our results were successfully validated against the presence of human WNV cases at two geopolitical units: towns in CT and counties in the Northeast United States. Together, we were able to utilize surveillance data on WNV's primary vector in the Northeast United States to develop an accurate and validated WNV risk projection pipeline that quantifies and accurately communicates the risks of WNV to humans in areas with and without mosquito surveillance. Our results confirm that detecting enzootic WNV transmission in the Northeast directly relates to risk of WNV to humans, which should encourage further support and development of robust arbovirus surveillance systems throughout the United States and abroad.

All training models selected final variables of known importance to WNV transmission and risk: the number of collected *Cx. pipiens* mosquitoes, factors associated with urbanization (human population density and percent intensely developed land cover), current and prior drought conditions, and current and prior weather patterns. The rank importance of *Cx. pipiens* collections in this model is not surprising, given a wealth of data supporting *Cx*. *pipiens* as the most important primary vector of WNV in the northeast. Thus, predicting abundance of *Cx. pipiens* mosquitoes across the region was an important component of our modeling framework. Compared to WNV training models, many more candidate variables were selected in training models of *Cx. pipiens* monthly abundance, which reflects prior results using the CAES surveillance data set showing more factors influence mosquito species distributions than arboviral species distributions (32). This may represent a key consideration when modeling WNV risk in areas in the United States with other WNV primary vectors, such as *Cx. tarsalis* in the western United States (44). Across candidate training models for *Cx. pipiens*, variables associated with average monthly temperatures, human population density, and prior drought conditions were commonly the three most important predictors. Despite these commonalities, the best fitting BRT model included three metrics of precipitation (current and prior drought conditions plus the difference in precipitation patterns compared to normal) in addition to human population density and wetland habitats. The inclusion of precipitation metrics possibly captures the important relationship between available larval habitats and productivity, and it should not be surprising that extremely wet conditions are associated with increased mosquito abundance. Increased rainfall is also related to increased dispersal of different *Culex* spp. in search of oviposition habitats, which could also explain increased collections and detections of WNV in gravid traps (45, 46). The inclusion of wetland habitat measures also probably reflects the design of the CAES surveillance system, which also surveys for EEEV in these habitats; the form of the relationship matches known ecologies, and the general trend in these variables was that the more wetland habitat, the fewer *Cx. pipiens* mosquitoes were collected. This small result further indicates that our model sufficiently distinguished between sites typical of WNV and sites representative of other arboviral systems.

The importance and constant inclusion of human population density across *Cx. pipiens* and WNV training models perhaps indicates that this variable more accurately reflects aspects of the built environment that provides important larval habitats for *Cx. pipiens* reproduction (i.e. catch basins, combined sewer overflows, and other storm water management systems) (47–49) compared to a general, categorical land cover classification. Previous studies have identified important relationships between *Cx. pipiens* population dynamics, WNV infection metrics, and presence of storm water management infrastructure (48, 50); future research in CT could examine these mosquito-infrastructures in more detail to identify mechanisms supporting increased collections of *Cx. pipiens* in more densely population human habitats. The high importance of human population density could also reflect the design of the CAES surveillance system, which tends to sample more frequently in the two dominant land cover classes in CT: forested or developed (32), with roughly equal distribution of sites in the training data between the two land cover classes. While these land cover distinctions in the Northeast may not extrapolate to other settings and contexts (38, 39, 43, 51), future modeling frameworks using a finer temporal and spatial grain which can better control for trapping effort combined with methods more sensitive to zero-inflated disease data may be better able to disentangle complex relationships between climate, urbanization, and WNV incidence that could be masked in our approach.

While no variables were included that specified the time of year (such as a monthly seasonality term) or directly identified the spatial location of a surveillance trap, all WNV risk projections predicted a greater magnitude and prevalence of WNV risk from July through September in the most urban of areas of CT and the Northeast; variance of WNV monthly detection probabilities was also highest during these periods and areas indicating that our model appropriately captured variation in weather conditions between transmission seasons. Qualitative inspections of seasonal risk maps further confirm that weather variability drives spatial expansions/contractions of high-risk areas—further distinguishing variation in risk among years.

Validating our risk projections against human case data in the Northeast proved successful even when controlling for human population size of a town or county. We believe this is an important advancement in the field, as our results point to a direct relationship between sylvatic activity and incidence of human spillover: the more likely WNV activity will be detected in mosquitoes, the more likely a human case will occur regardless of human population size. Other metrics of human populations, such as demography, social inequity, and access to healthcare are other important metrics to consider when predicting incidence of WNV in humans (52–54); these variables can also influence patterns of mosquito community composition and collections (55, 56). Future studies that examine these relationships may refine the predicted incidence of WNV in mosquitoes and humans.

Other studies have identified lags in extreme events or used measures of climatic variability as important predictors of *Cx. pipiens* collections (9, 57, 58). Many of these studies’ objectives focused on seasonal variation in WNV transmission intensity, utilized finer temporal units, and some relied on more sophisticated methodologies. Although extreme events are difficult to forecast, they can have greater proportional impacts in urban spaces, especially when it comes to extreme heat. Such results would not change the primary message of our study, which is the probability of detecting WNV-infected *Cx. pipiens* mosquitoes is highest in urbanized spaces during the hottest months of the year. However, given that weather data are more often freely accessible online and captured at greater frequencies than land cover and human population density estimates, more detailed analyses of weather data sources for mosquito species distributions may prove a more powerful approach to projecting VBD risk (59).

One component missing from our risk estimation pipeline concerns evidence of WNV in secondary vectors and in avian reservoir hosts. Prior work with the CAES surveillance data set has identified *Cx. restuans*, *Cx. salinarius*, and occasionally *Culiseta melanura* as important secondary vectors of WNV in CT (32). We suspect that methods that project risk into *Cx. restuans* and *Cx. salinarius* would mostly identify similar risk factors as for *Cx. pipiens* based on prior analyses of CAES data. Analyses of *Cs. melanura* could identify expansions of risk later in the summer into nonurbanized spaces (see Ref. (32) for WNV detection trends in *Cs. melanura*). This is based on prior knowledge of *Cs. melanura* in CT (60, 61), though future studies of WNV infections in all three species, as well studies into the relationships between vector community composition and WNV transmission, are needed to determine the exact associations between collections of multiple species and WNV detections. Nevertheless, developing other species-specific models for secondary WNV vectors such as *Cx. restuans* and *Cx. salinarius* could refine predicted WNV activity and identify important land cover classes linked to WNV activity (32, 62).

## Conclusions

Together, the primary risk factors for detecting a human case of WNV in any area in the northeast United States include human population size (e.g. more opportunities for a case to occur as well as an indication of urbanization) and a high probability of WNV activity in mosquitoes. Active mosquito-based WNV surveillance remains the gold standard for assessing the risk of WNV activity in mosquitoes and should be utilized wherever possible to inform the public about the risk of WNV. This is because surveillance provides early evidence of virus activity as well as information on the abundance, distribution, identity, and infection rates of potential mosquito vectors. Nevertheless, mosquito surveillance is labor intensive and is limited by practical constraints on the number of locations that can be sampled. Our presented methods provide a means to overcome this limit of spatial surveillance by providing the ability to accurately predict seasonal risk of WNV in unsampled areas. These predictions can be incorporated to WNV risk forecasts that are presented together with weather and pollen/allergies forecasts as is being done in some major US cities (63). This integrated approach increases the capability of CAES and other WNV surveillance programs in the Northeast to effectively serve the public by communicating WNV risk in regions that are and are not monitored by traps. We encourage other districts throughout the United States and abroad to implement similar approaches using their own unique data sets to extend their abilities to communicate risk in (un)sampled spaces.

## Supplementary Material

pgaf227_Supplementary_Data

## Data Availability

All raw data are archived in Mendeley Data using the following DOI: 10.17632/6v43bvt7mj.1. Any visualized and presented human data are freely available from online sources. Additionally, no identifiers are provided.

## Preprints

A prior version of this manuscript is posted on a preprint: https://doi.org/10.21203/rs.3.rs-3293255/v1.
