# Supplementary material for: Using mosquito and arbovirus data to computationally predict West Nile virus in unsampled areas of the Northeast United States
Source: PNAS Nexus. 2025 Aug 19;4(8):pgaf227. doi: 10.1093/pnasnexus/pgaf227 (PMC12362355; doi:10.1093/pnasnexus/pgaf227)
Supplement: pgaf227_Supplementary_Data [file pgaf227_supplementary_data.pdf]

Supporting Methods for

Title: Using mosquito and arbovirus data to computationally predict West Nile virus in unsampled areas of the Northeast United States

Authors: Joseph R. McMillan[1], James Sun[2], Luis Fernando Chaves[3], Philip M. Armstrong[4]

Author affiliations:

[1]Department of Biological Sciences, Texas Tech University, Lubbock, TX, USA

[2]Clark Scholars Program, Department of Biological Sciences, Texas Tech University, Lubbock, TX, USA

[3]Department of Environmental and Occupational Health, School of Public Health and Department of Geography, Indiana University, Bloomington, IN, USA

[4]Department of Entomology, The Connecticut Agricultural Experiment Station, New Haven, CT, USA

Corresponding Author:

Joseph R. McMillan

Biology Building, Rm 212

2901 Main Street

Lubbock, TX 79404

josmcmil@ttu.edu

ORCID 0000-0002-6909-950x

Classification: Public Health and Epidemiology

Keywords
machine learning, hierarchical modeling, West Nile virus, surveillance, *Culex pipiens*, risk mapping

Mosquito and arbovirus surveillance data: Mosquito and WNV data were obtained using hay-lactalbumin infusion-baited gravid traps at 87 surveillance sites in CT from 2001 – 2020. These traps are the most efficient surveillance tool for detecting WNV-infected *Cx. pipiens* mosquitoes (1), which is the primary enzootic and epidemic vector of WNV in the US northeast (2, 3). Briefly, sites were sampled by operating traps overnight on a 10-day rotation each summer from June through October, and all female mosquitoes were identified to species using a dichotomous key (4) and tested for nine arboviruses using viral cell culture and RT-PCR techniques (5). Collection sites were sampled more frequently (1-2 times weekly) for the remainder of a season if mosquitoes tested positive for an arbovirus of primary public health concern (WNV or EEEV); gravid traps are employed in all supplemental sampling events regardless of if the arbovirus under investigation is WNV or EEEV.

Prior work with the CAES dataset revealed that aggregating mosquito and arbovirus collections to the scale of a week resulted in a majority of zero values for WNV detection at any given site for all species; thus, for our modeling purposes we chose to aggregate mosquito collections and WNV detections to the scale of a month. Monthly *Cx. pipiens* collections were corrected for the number of sampling events that took place at a site during a given month; these values were then rounded to the nearest whole number for model training purposes. For WNV detections, prior explorations with the data examined multiple response forms: (un)corrected total number of positive pools, minimum infection rates (MIRs), and (un)corrected number of WNV positive trap nights. In all instances, these response forms generated an overabundance of zero values or presented a numerical form inconsistent with the error

distributions available in the gradient boosting machine model R packages we describe later in the report. WNV detection forms that could be modeled (such as number positive pools and MIRs) rarely predicted WNV detections accurately (i.e., models predicted where WNV was not found and generally never predicted WNV detections). Thus, to create a strong enough signal to be predicted from the data, our calibration models of WNV detection modeled detection as 1 – at least one trap night per month contained WNV positive mosquitoes and 0 – no trap nights contained WNV positive mosquitoes per month. This approach ultimately generates a model that is trained to recognized patterns associated with (in)consistent WNV detection events (presumably, if focal WNV transmission events are intense, they will be repeatedly detected over multiple trap nights, thus increasing the probability that a site is labeled as 1 – positive). Given our ultimate objective is to map spatial risk of WNV activity across CT, this approach is appropriate.

Weather data: Monthly average temperature and precipitation records from 2001 to 2020 as well as monthly climate 30-year normals were obtained from PRISM (https://prism.oregonstate.edu/) (6) by extracting data to the specific surveillance site coordinates using the Data Explorer tool (https://prism.oregonstate.edu/explorer/). PRISM uses a default 4km grid system, meaning climate values were assigned to points based on the grid in which they are located. Because the CT surveillance program only operates traps from June to October, we extracted the values for each site that aligned with the mosquito collection period plus a one-month lag (e.g., May through

October). We calculated the absolute difference in the observed and normal values for these time intervals.

Monthly drought conditions, defined using the Palmer Drought Severity Index (PDSI), were obtained from the National Oceanic and Atmospheric Administration. PDSI is a composite score of dryness and is reported on a standardized scale of -10 (extremely dry) to +10 (extremely wet), and NOAA reports PDSI metrics at multiple geopolitical and environmental units, including county, state, and climate zone levels. For the purposes of our modeling work, we extracted PDSI values to the point level based on NOAA climatic zones. These records are not available as maps, such that to match the PDSI value to a CAES collection site, we first had to assign collections sites to their respective climate zone. We then joined PDSI data to collection sites based on climate zone and month of collection. In all training models, we included the current month and a 1-month lag in PDSI.

Because our mosquito data was aggregated to the level of a month and PRISM and NOAA provides easily accessible monthly weather reports and maps, we chose to include only a 1-month lag in our weather variables in our modeling efforts and did not consider other climate variables beyond temperature, precipitation, and drought conditions.

Landcover data: Landcover data was obtained from the Multi-Resolution Land Characteristics Consortium. Briefly, this site hosts data on annual coverage of over sixteen land cover class estimates across the United States at annual intervals. Land cover classes in Connecticut are as follows: open water, developed < 25%, developed >

25% & < 50%, developed > 50% & < 75%, developed > 75%, barren, deciduous forest, coniferous forest, mixed forest, scrub, grass, pasture, cultivated crops, wetland forested, and wetland emergent. At time of analysis, not all annual years were readily available from MRLC; our training models utilized data estimated from 2001, 2004, 2006, 2008, 2011, 2013, 2016, and 2019. We modeled land cover as the percent land cover of each type within 5 km of a surveillance site. To build a suite of land cover variables for model training, raster files for all listed years were downloaded and transformed to match the CRS of CAES surveillance coordinates. We then extracted the number of pixels of each land cover type within 4 km x 4 km raster cells and then recalculated percent land cover based on the total number of pixels within a cell. We then extracted the average percent land cover for each type

Human population density data: Because numerous field studies in the eastern and Midwestern US identify urban habitats as important metrics of WNV risk in mosquitoes and humans, we included human population density data as an additional indicator of urbanization in our calibration models. While human population density alone may not fully capture some of the nuances of urban infrastructure associated with *Cx. pipiens* population dynamics (such as catch basin density and/or percent impervious surface), it was assumed that these metrics are likely correlated, and population density sufficiently captures metrics of US urban infrastructure.

We downloaded the gridded global population density raster image for 2020 from Columbia University's Socioeconomic Data and Applications Center (https://sedac.ciesin.columbia.edu/data/set/gpw-v4-population-density-adjusted-to-

2015-unwpp-country-totals-rev11/data-download), cropped the image to match the spatial extent of CT, resampled the image to match the 4x4km resolution of the PRISM data, then extracted the average population density estimates within 5km of each surveillance site.

Model development. All training models utilized monthly variables. Response variables were either *Cx. pipiens* monthly trap night collections or WNV detection (1 – one or more positive trap nights in a month, 0 – no positive trap nights in a month). The full list of predictor variables is available in **Table 1** in the main manuscript.

We used three types of modeling approaches to calibrate models of *Cx. pipiens* monthly trap night collections and WNV monthly detections. We first used gradient boosting machines, specifically boosted regression trees (BRT), in the “dismo” and “gbm” R packages (7) as our primary modeling approach. All processed CAES surveillance, weather, land cover, and human population density data were randomly assigned as training and test data using a 70/30 training/test ratio. Full models included all fifteen land cover classes, a subset of monthly and 1-month lag weather variables, and human population density within 5 km of a surveillance site; full models of WNV detection (1 – one or more positive trap nights in a month, 0 – no positive trap nights in a month) included the same variables with the addition of the observed *Cx. pipiens* monthly trap night collection.

For all candidate training models, we first compared full models containing a subset of pairwise combinations of the monthly weather variables. In these subsets, only one monthly weather form (average or difference from normal) or weather variable

(temperature or precipitation) was used. We simplified these training models through sequential elimination of the variables with lowest influence; the *gbm* package allows for model simplification up to X-2 variables and explained deviance is calculated with each additional variable removal. The best performing model for each response variable was assessed from the vantage points of model deviance, root mean square error (RMSE, assessed using the test data), and parsimony (i.e., fewest variables). Once all BRT models were assessed, we compared their performance a random forest regression (*Cx. pipiens* collections), classification (WNV detection), and an all pairwise interaction general linear model (GLM) using the same final terms as in the BRT models. Each RF and GLM training models was further simplified using backward selection. RF model simplification works similarly to BRT models, and RF simplification was performed using functions available in the randomForest and claret packages with variables removed based on importance scores. GLM backward selection procedures eere based on AIC. The use of BRT and GLM models was done to compare their ability (or lack of ability) to capture non-linear relationships, respectively. Final models utilized for validation were chosen based on lowest RMSE of the simplified BRT and GLMs.

Predicting WNV detection probabilities in CT and New England.

<u>Connecticut Towns:</u> To generate monthly predictions of WNV detection probabilities in *Cx. pipiens* mosquitoes, we first had to generate statewide raster data sets of all training model variables. We first defined a 4 km x 4 km raster grid at the spatial extent of the state of CT. For weather variables, we downloaded the temperature/precipitation raster files from PRISM that matched the forms of the final models for the Years 2001 to 2022

using the “prism” R package, transformed the weather data CRS, cropped the images to match the extent of CT, then resampled the image based on the defined 4x4 km raster grid. This was performed using a combination of functions available in the “sf” and “raster” packages (8, 9); we used the same methodology to generate a raster image of human population density for the years 2000, 2005, 2010, 2015, and 2020. Land cover files were generated as listed above. As drought data was only available in tabular form, we first downloaded the GIS shapefiles for US climate zones from NOAA then joined all monthly PDSI data to those shapefiles. We then rasterized the statewide monthly values using functions available in the “terra”, “sf”. And “raster” R packages. To generate predicted *Cx. pipiens* monthly trap night collections throughout CT, we created a RasterStack of all variables in the best performing training models and then predicted collections in each raster cell using the built-in prediction function in the “raster” package for both BRT- , RF-, and GLM-based models. The predicted *Cx. pipiens* monthly trap night collections raster layer was then added to the RasterStack containing the variables in the final WNV model, and WNV detection probabilities were generated for each raster cell in CT using the final WNV models and the predict function in “raster”.

Northeast Counties: We only examined relationships between county level predicted WNV activity in mosquitoes and annual human WNV case counts (inclusive of all reported infections – neuro-invasive, non neuro invasive, and blood donors) for the years 2021 and 2022. Land cover data at the scale of the northeast – inclusive of Connecticut, Maine, Massachusetts, New Hampshire, New Jersey, New York, Rhode Island, and Vermont – first had to be downloaded at the US continent scale for the Year

2021. Then, to speed up processing the image at 4 x 4 km raster cells, we first clipped the image into each individual State; we then used the methodology above to calculate percent land cover for each variable type. To match this process, weather and population density data were also extracted/processed at the extent of each state. Once state-level data were processed, we used the same prediction framework listed above to generate WNV detection probabilities throughout each state.

Model validation.

Connecticut Towns: We compared the predicted probability of WNV monthly detections in mosquitoes from our final candidate models to the observed presence/absence of WNV detections in humans in CT 2001 – 2020. To do this, we first extracted the average monthly raster-based WNV detection probability to the level of the administrative unit of CT towns using the extract function in the “raster” package (8). Shapefiles for the political boundaries of CT were downloaded from (https://ct-deep-gis-open-data-website-ctdeep.hub.arcgis.com/search?groupIds=71c5c4a9c6ea4ea8ab54d1bf1faaeed8). We converted this file into a data frame and then joined the data to reported human case totals for each town. We used binomial-error generalized mixed effects models with observed WNV detections in humans (modeled as detected 1, not detected 0) at the town level as a response variable, the average monthly predicted WNV mosquito detection probability as fixed effects, the town’s human population size (log transformed) as an intercept offset, and town name and year of collection as a random effects (to control for repeated observations (10)). We chose human case occurrence

rather than absolute number of cases since there were rarely more than 1 human case for any given year and town combination.

Northeast Counties: We repeated the above exercise yet for northeast counties; county shape files were accessed using the “maps” R package. We then combined all state level data frames and joined data on the observed number of human cases reported to CDC’s Arbonet 2021 - 2022. Because CDC reports human infections of WNV at an annual scale, we calculated the average and maximum monthly and county level WNV detection probability in mosquitoes for each year as our primary predictor term. Because the range human population densities across counties in the northeast was considerably greater than that of CT alone, we used zero-inflated GLMs in the pscl R package. In these models, presence/absence of a human case in a county was modeled as a function of human population density while the number of human cases was modeled as a function of WNV detection probabilities in mosquitoes. The underlying error distribution of case counts in these models was modeled using a negative binomial distribution.

References

1. Reiter P. A portable battery-powered trap for collecting gravid Culex mosquitoes. Mosq News. 1983;43(4):496-8.

2. McMillan JR, Armstrong PM, Andreadis TG. Patterns of mosquito and arbovirus community composition and ecological indexes of arboviral risk in the northeast United States. PLoS neglected tropical diseases. 2020;14(2):e0008066. Epub 2020/02/25. doi: 10.1371/journal.pntd.0008066. PubMed PMID: 32092063; PMCID: PMC7058363.

3. Andreadis TG. The contribution of Culex pipiens complex mosquitoes to transmission and persistence of West Nile virus in North America. Journal of the American Mosquito Control Association. 2012;28(4 Suppl):137-51. Epub 2013/02/14. doi: 10.2987/8756-971X-28.4s.137. PubMed PMID: 23401954.

4. Darsie R, Ward R. Identification and geographical distribution of the mosquitoes of North America, north of Mexico: University Press of Florida; 1981.
5. Armstrong PM, Andreadis TG, Finan SL, Shepard JJ, Thomas MC. Detection of infectious virus from field-collected mosquitoes by vero cell culture assay. J Vis Exp. 2011(52). Epub 2011/06/23. doi: 10.3791/2889. PubMed PMID: 21694689; PMCID: PMC3197060.
6. Daly C, Halbleib M, Smith JI, Gibson WP, Doggett MK, Taylor GH, Curtis J, Pasteris PP. Physiographically sensitive mapping of climatological temperature and precipitation across the conterminous United States. International Journal of Climatology: a Journal of the Royal Meteorological Society. 2008;28(15):2031-64.
7. Elith J, Leathwick JR, Hastie T. A working guide to boosted regression trees. Journal of animal ecology. 2008;77(4):802-13.
8. Hijmans RJ, Van Etten J, Mattiuzzi M, Sumner M, Greenberg J, Lamigueiro O, Bevan A, Racine E, Shortridge A. Raster package in R. Version https://mirro rs sjtug sjtu edu cn/cran/web/packa ges/rast e r/raster pdf. 2013.
9. Pebesma E. Simple Features for R: Standardized Support for Spatial Vector Data. R J. 2018;10(1):439-46. PubMed PMID: WOS:000440997400030.
10. Chaves LF. An entomologist guide to demystify pseudoreplication: data analysis of field studies with design constraints. Journal of medical entomology. 2010;47(3):291-8. Epub 2010/05/26. doi: 10.1603/me09250. PubMed PMID: 20496574.

Supporting Figures for

Title: Using mosquito and arbovirus data to computationally predict West Nile virus in unsampled areas of the Northeast United States

Authors: Joseph R. McMillan[1,2], James Sun[2], Luis Fernando Chaves[3], Philip M. Armstrong[4]

Author affiliations:

[1]Department of Biological Sciences, Texas Tech University, Lubbock, TX, USA

[2]Clark Scholars Program, Department of Biological Sciences, Texas Tech University, , Lubbock, TX, USA

[3]Department of Environmental and Occupational Health, School of Public Health and Department of Geography, Indiana University, Bloomington, IN, USA

[4]Department of Entomology, The Connecticut Agricultural Experiment Station, New Haven, CT, USA

Corresponding Author:

Joseph R. McMillan

Biology Building, Rm 212

2901 Main Street

Lubbock, TX 79404

josmcmil@ttu.edu

ORCID 0000-0002-6909-950x

Classification: Public Health and Epidemiology

Keywords
machine learning, hierarchical modeling, West Nile virus, surveillance, *Culex pipiens*, risk mapping

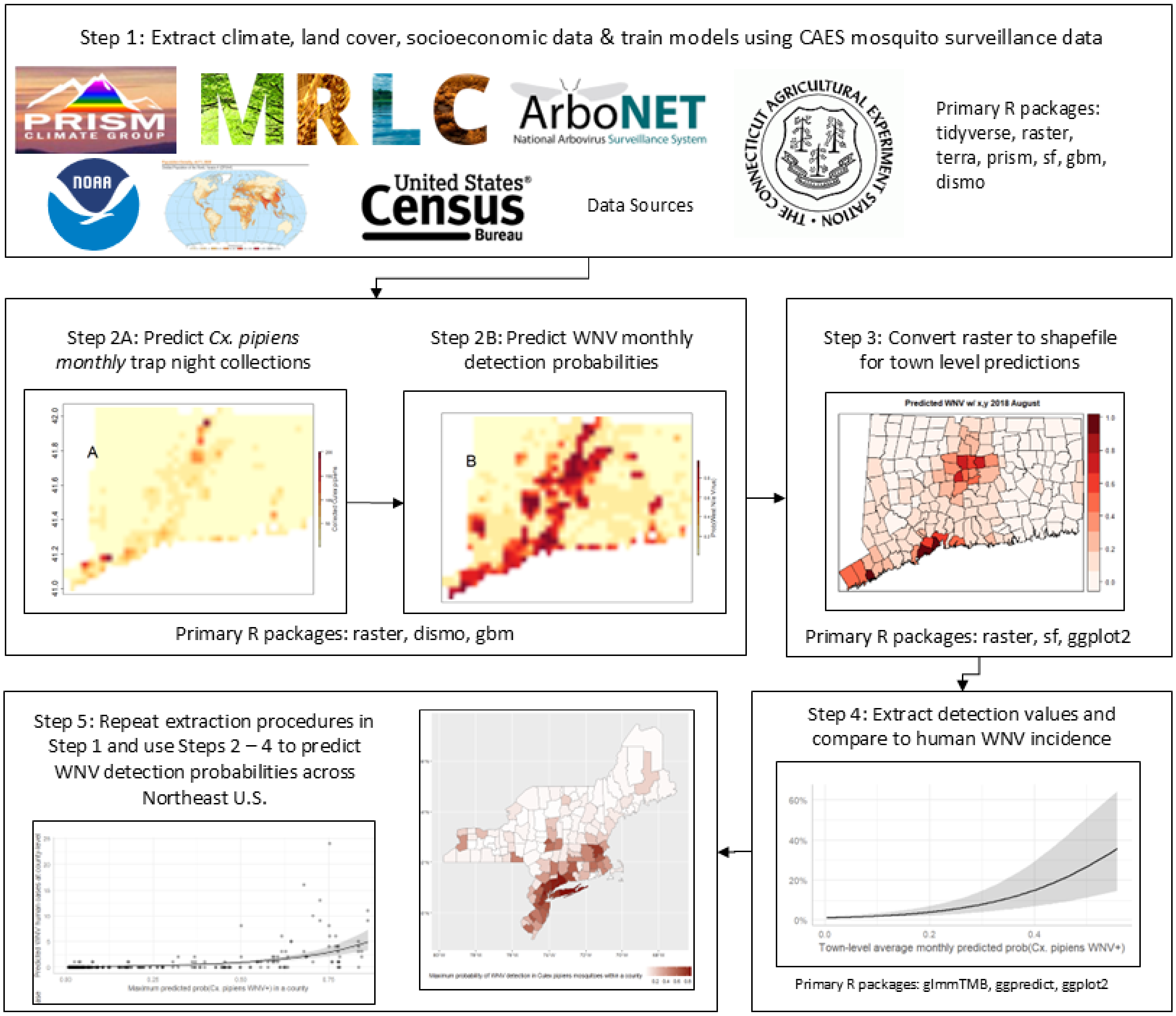

Supplementary Figure 1. Graphical abstract of project workflow.

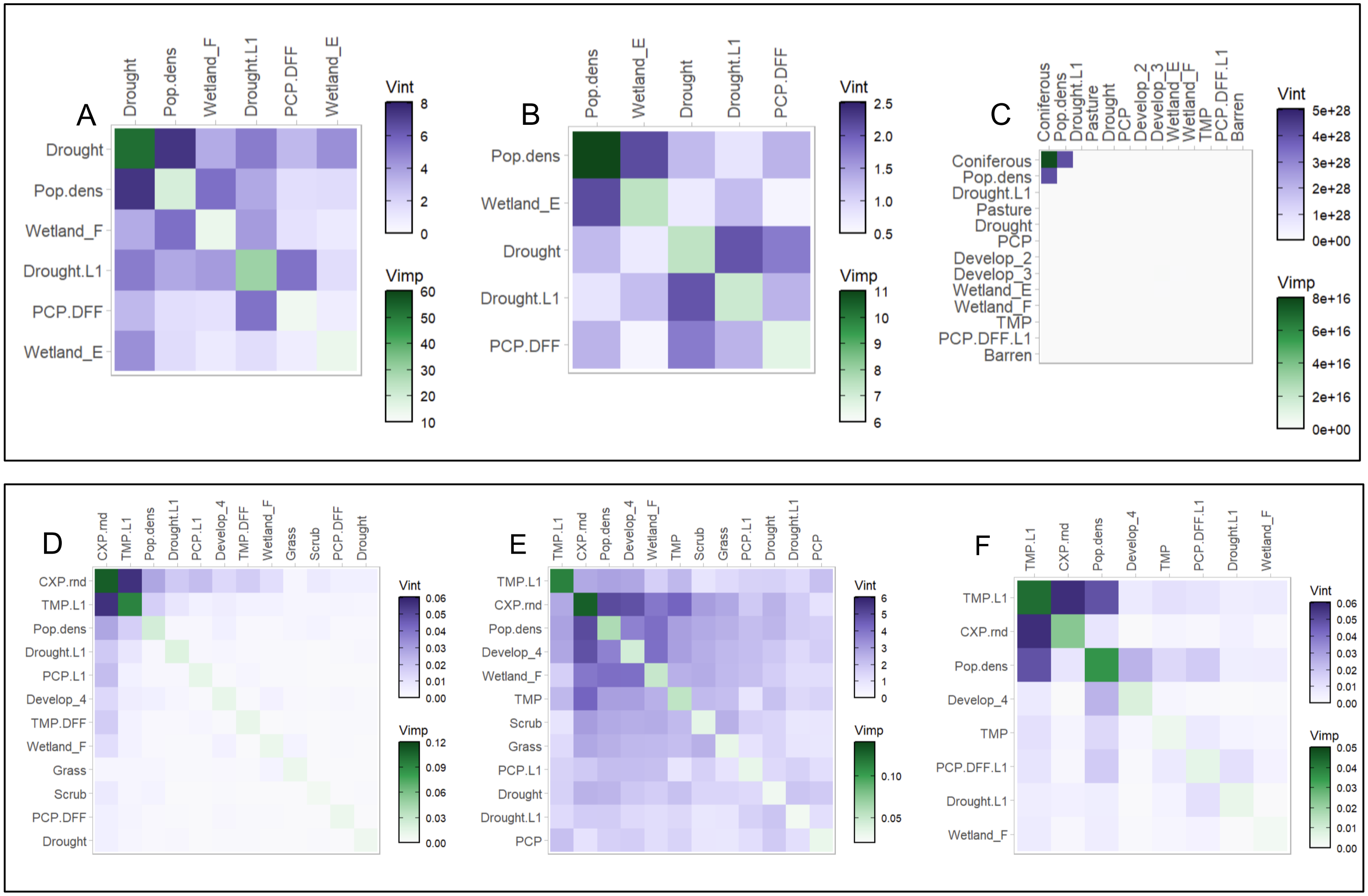

Supplementary Figure 2: Variable network plots from the best fitting simplified boosted regression tree (A, C), random forest (B, D), and generalized linear model (C, F) of monthly *Culex pipiens* mosquitoes (A – C) and West Nile virus detection probabilities (D – F) from mosquitoes captured in gravid traps at 87 sites in Connecticut, USA from 2001 – 2020. Green colors indicate ranked variable importance while purple colors indicate ranked variable interactions. Intensity of color indicates magnitude of importance or strength of interactions. Network plots generated using R package vivid.

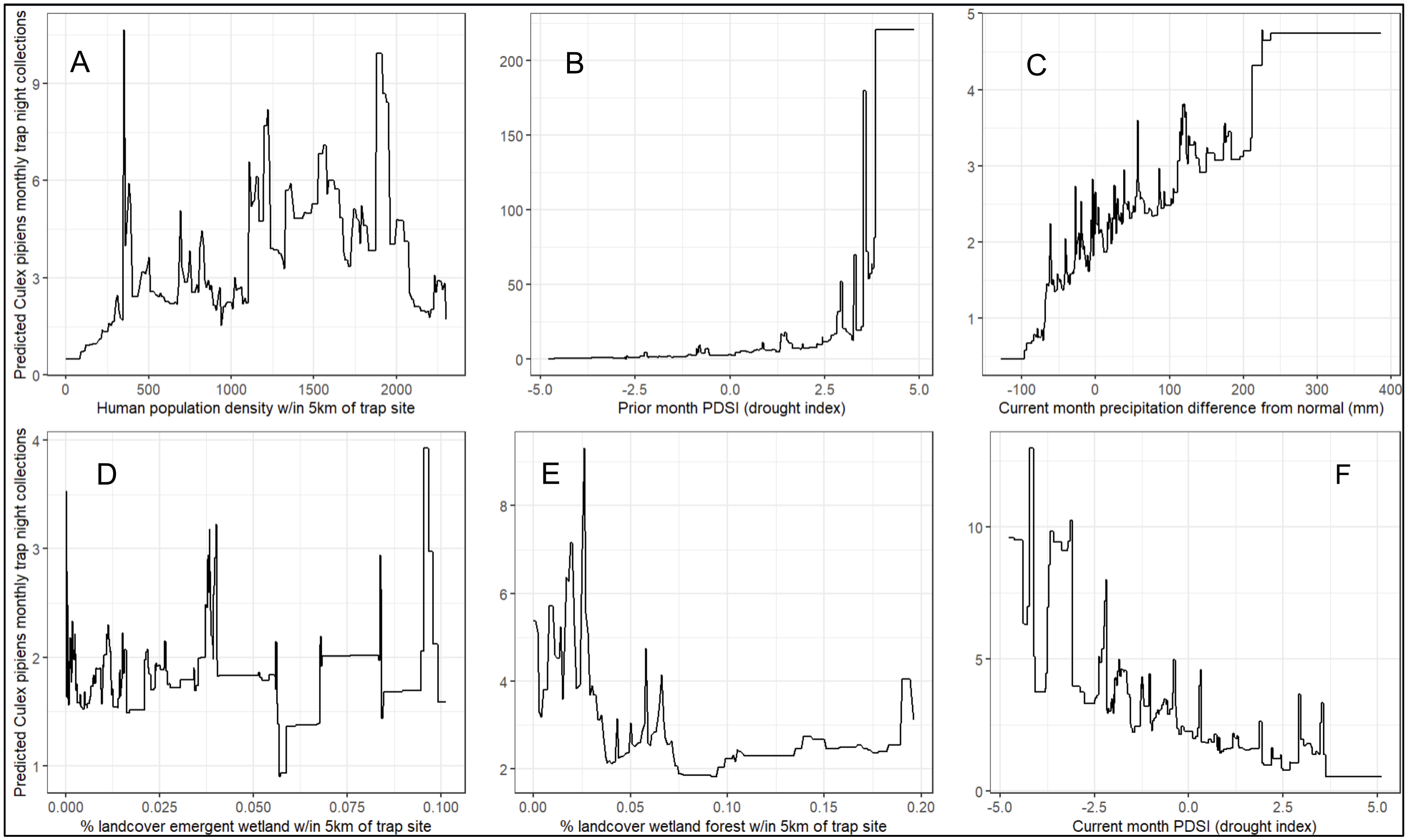

Supplementary Figure 3. Partial dependence plots from variables included in the best fitting simplified boosted regression tree of monthly *Culex pipiens* mosquitoes captured in gravid traps at 87 sites in Connecticut, USA from 2001 – 2020. In all panels, the y-axis is the predicted monthly trap night collection of *Cx. pipiens* mosquitoes. X-axes the variables included in the best performing BRT: A) human population density, B) prior month Palmer Drought Severity Index, C) current month's precipitation difference from normal, D) percent emergent wetland land cover within 5km of a trap sites, E) present forested wetland within 5km of a trap sites, and F) current month's PDSI values. In all panels, lines represent the predicted relationship between the predictor (x axis) and response variable (*Cx. pipiens* monthly trap night collections) while holding all other variables in the model constant.

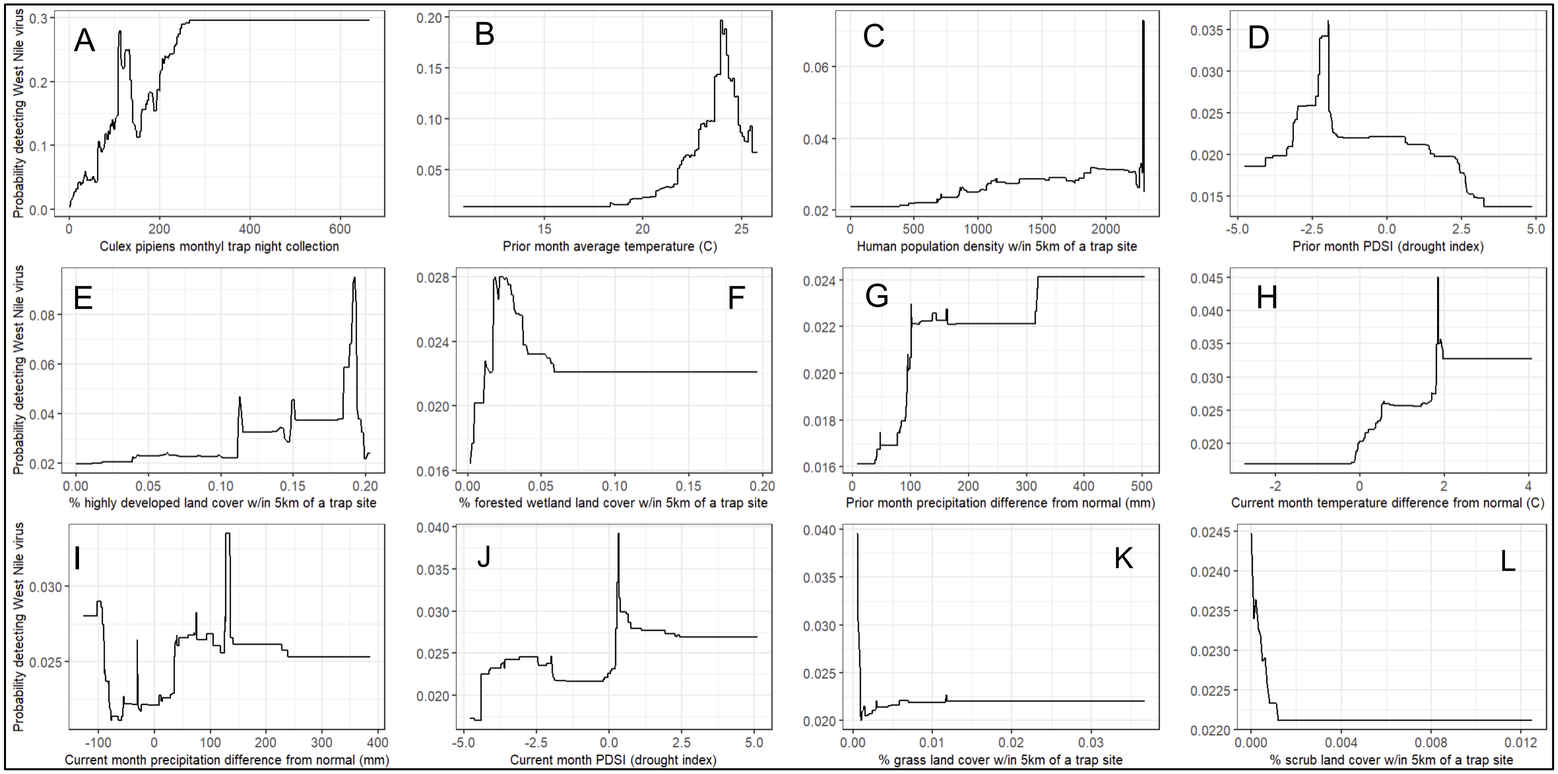

Supplementary Figure 4. Partial dependence plots from variables included in the best fitting simplified boosted regression tree of monthly West Nile virus detection probabilities in *Culex pipiens* mosquitoes captured in gravid traps at 87 sites in Connecticut, USA from 2001 – 2020. In all panels, the y-axis is the predicted monthly detection of WNV in *Cx. pipiens* mosqitoes. X-axes the variables included in the best performing BRT: A) *Cx. pipiens* monthly trap night collections, B) prior month average temperature, C) human population density, D) prior month Palmer Drought Severity Index, E) percent highly developed land cover, F) percent forested wetland land cover, G) prior month's precipitation difference from normal, H) current months temperature difference from normal, I) current month's precipitation differcne from normal, J) current month's PDSI, K) percent grass land cover, and L) percent scrub land cover within 5km of a trap sites. In all panels, lines represent the predicted relationship between the predictor (x axis) and response variable (*Cx. pipiens* monthly trap night collections) while holding all other variables in the model constant.

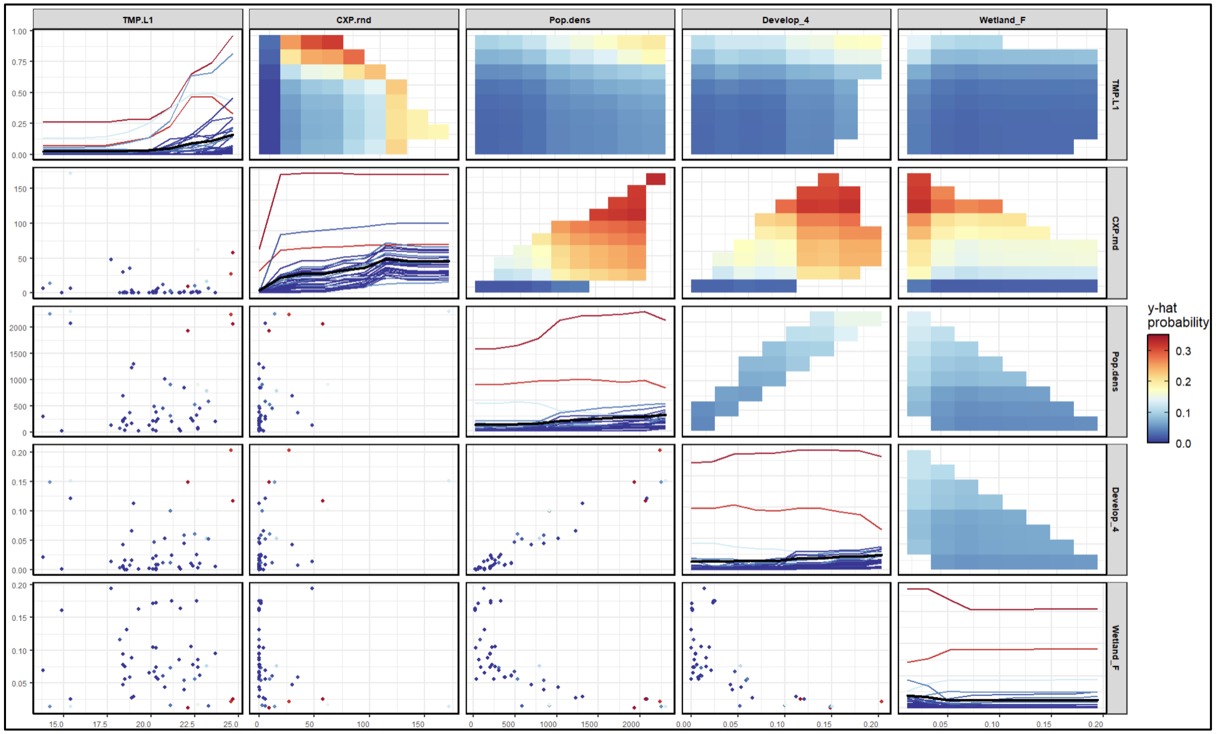

Supplementary Figure 5: Generalized pairs partial dependence (pdp) and individual conditional expectations (ICE) plot from the top five variables from the best fitting simplified random forest classification model of West Nile virus presence/absence in *Culex pipiens* mosquitoes captured at 87 sites in Connecticut, USA from 2001 – 2020. Plots generated using the vivid R package pdpPairs() function, and the fitted relationships are considered agnostic representations of black-box type models (such as random forests). pdp/ICE plots provide visual model assessments of fitted relationships as a function of individual instances within the training data. The diagonal shows the univariate relationship between the listed variable and the probability of WNV detection (TMP.L1 – one month lag in average temperature, CXP.rnd – monthly *Cx. pipiens* trap night collection, Pop.dens = human population density within 5km of a trap site, Develop_4 = % land cover classified as highly developed within 5 km of a trap site, and Wetland_F = percent land cover classified as forest wetland within 5 km of a trap site). Lines, points, and colored squares represent the ICE for 30 randomly selected instances within the data set. The partial dependence between two variables is shown in the upper triangle with colors representing the predicted detection of WNV as a function of the two variables. The lower triangle shows the ICE data points. Low classification probabilities (represented as color gradients in the y-hat probability legend) indicate that random forest interactions of the training models were poor predictors of WNV detections.

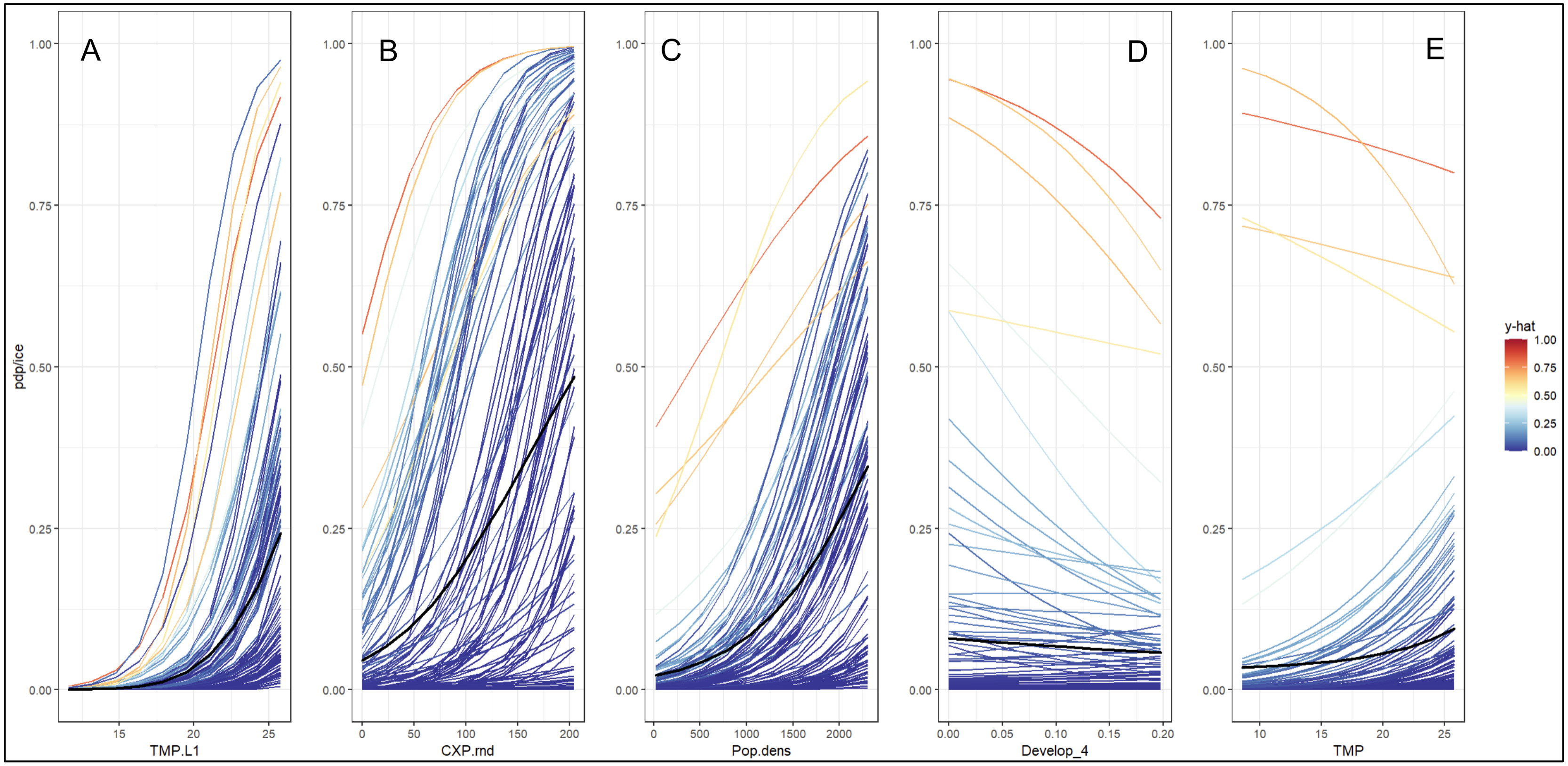

Supplementary Figure 6: Partial dependence (pdp) and individual conditional expectations (ICE) plot from the top five variables from the best fitting generalized linear model of West Nile virus presence/absence in *Culex pipiens* mosquitoes captured at 87 sites in Connecticut, USA from 2001 – 2020. Plots generated using the vivid R package using the pdpVars() function. pdp/ICE plots provide visual model assessments of fitted relationships as a function of individual instances within the training data. In all panels, the y-axis indicates the probability of a monthly WNV detection in *Cx. pipiens* mosquitoes. Lines represent fitted relationships from 30 randomly selected instances within the data set. The solid black line in each plot represents the average predicted relationship. X-axis are as follows: TMP.L1 = one month lag in average temperature, CXP.rnd = monthly *Cx. pipiens* trap night collections, Pop.dens = human population density within 5km of a trap site, Develop_4 = percent land cover classified as highly developed within 5km of a trap site, and TMP = average monthly temperature. While glms training models provided robust fit to the data, drastically variable ICE plots as shown for Develop_4 and TMP indicate overly complex interactions were prevalent in these model forms.

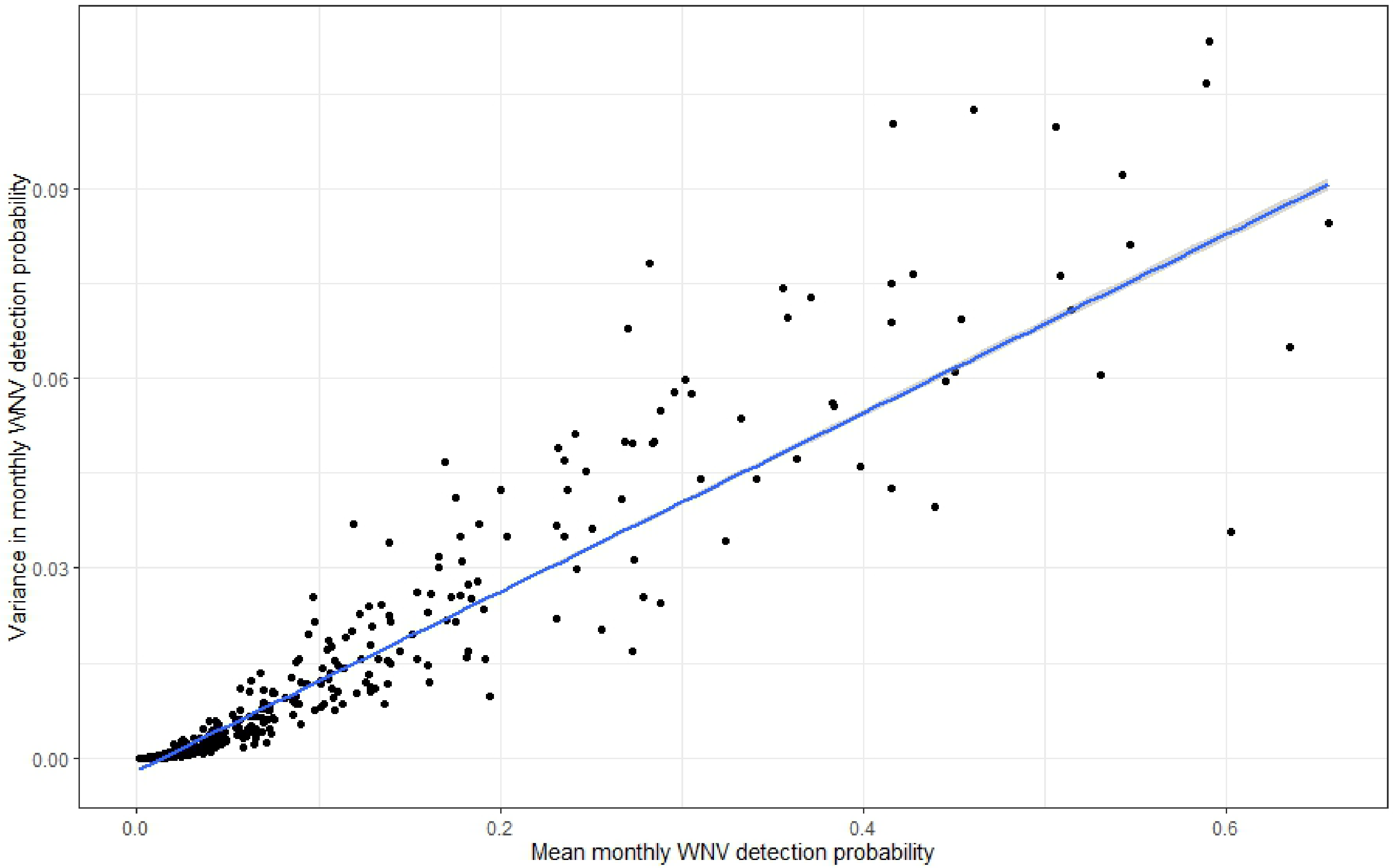

Supplementary Figure 7: Mean by variance plot for town-level monthly West Nile virus detection probabilities in *Cx. pipiens* mosquitoes in CT from 2001 – 2022.

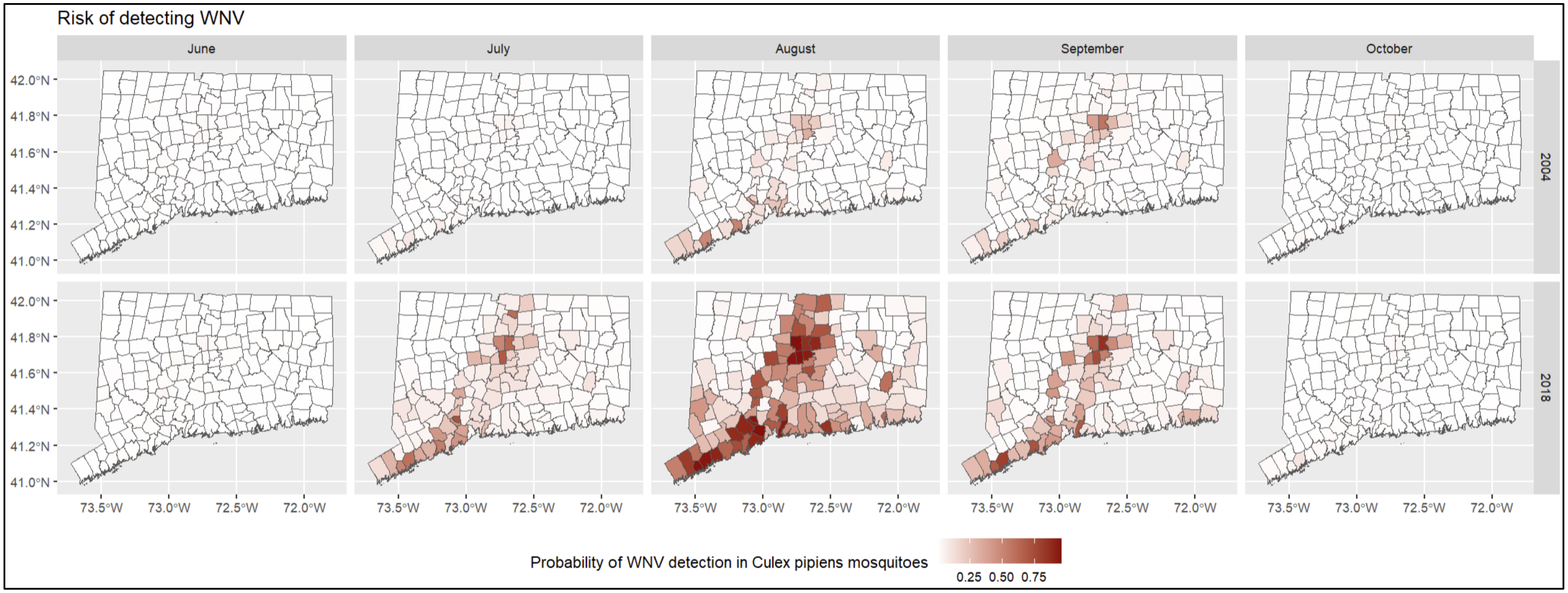

Supplementary Figure 8: Example risk maps based on a low West Nile incidence year (e.g., 2004) and a high WNV incidence year (2018). Rows represent years (2004 or 2018) and columns represent months (June – October). Colors represent the magnitude of the predicted WNV detection probability.

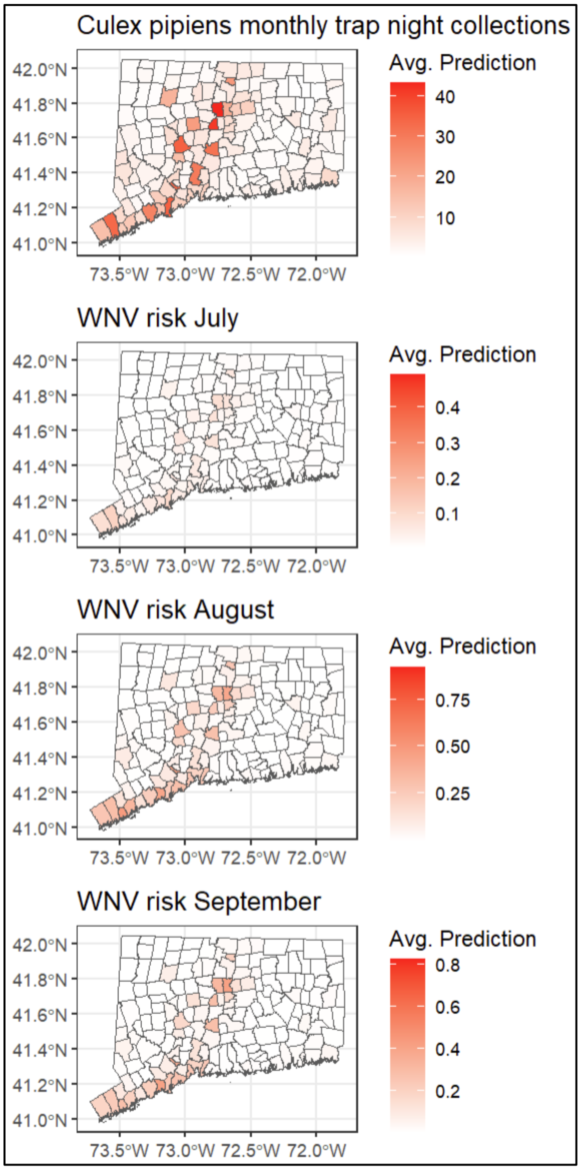

Supplementary Figure 9: Example risk plot created with user-specified values to demonstrate the modeling tool's ability to generate future spatial risk projections of West Nile virus in Connecticut, US. For weather variables that were either indexed (PDSI) or represented a difference from normal, we set all null values to 0. For weather variables that were an observed monthly value, we set null values to the specific month's normal value in the historical record. The top row provides an example of the *Cx. pipiens* monthly trap night collections for the month of August. The remaining rows show WNV detection probabilities for July through September as these are the highest risk months for WNV in CT. Rows represent years represent months (June – October). Colors represent the magnitude of the predicted *Cx. pipiens* trap night collection (top row) and WNV detection probabilities (remaining rows).

Supporting Tables For:

Title: Using mosquito and arbovirus data to computationally predict West Nile virus in unsampled areas of the Northeast United States

Authors: Joseph R. McMillan[1,2], James Sun[2], Luis Fernando Chaves[3], Philip M. Armstrong[4]

Author affiliations:

[1]Department of Biological Sciences, Texas Tech University, Lubbock, TX, USA

[2]Clark Scholars Program, Department of Biological Sciences, Texas Tech University, Lubbock, TX, USA

[3]Department of Environmental and Occupational Health, School of Public Health and Department of Geography, Indiana University, Bloomington, IN, USA

[4]Department of Entomology, The Connecticut Agricultural Experiment Station, New Haven, CT, USA

Corresponding Author:

Joseph R. McMillan

Biology Building, Rm 212

2901 Main Street

Lubbock, TX 79404

josmcmil@ttu.edu

ORCID 0000-0002-6909-950x

Classification: Public Health and Epidemiology

Keywords
machine learning, hierarchical modeling, West Nile virus, surveillance, *Culex pipiens*, risk mapping

List of 15 land cover variables (as %)	Weather variables	Socioeconomic variables
Water<br>Develop 1<br>Develop 2<br>Develop 3<br>Develop 4<br>Barren<br>Forested – deciduous<br>Forested – coniferous<br>Forested – mixed<br>Scrub/shrub<br>Grass<br>Pasture<br>Crop<br>Wetland – forested<br>Wetland – emergent	Temperature and Precipitation<br>1. All averages<br>2. Current month: averages/ prior month: difference from normal<br>3. Current month: difference from normal/ prior month: averages<br>4. All difference from normal<br>5. Temperature: averages/ precipitation: different from normal<br>6. Temperature: difference from normal/ precipitation: averages<br><br>Drought Conditions (PDSI) – current and prior month	Human population density w/in 5km of a trap site

Supplementary Table 1: Full list of variables used to train models of monthly *Culex pipiens* trap night collections and West Nile virus detection probabilities.

Variable List	WNV BRT	WNV RF	WNV GLM	CXP BRT	CXP RF	CXP GLM
% open water	0	0	0	2/6	1/2	2/2
% Develop 1	0	0	0	3/6	2/3	3/3
% Develop 2	0	0	0	4/6	3/4	4/4
% Develop 3	0	0	0	4/6	3/4	4/4
% Develop 4	6/6	3/6	6/6	2/6	2/2	2/2
% Barren	0	0	0	5/6	3/5	5/5
% Forested – deciduous	1/6	0	1/1	1/6	1/1	1/1
% Forested – coniferous	0	0	0	5/6	5/5	5/5
% Forested – mixed	0	0	0	1/6	0/1	1/1
% Scrub/shrub	2/6	2/2	2/2	1/6	1/1	1/1
% Grass	2/6	2/2	2/2	3/6	3/3	3/3
% Pasture	0	0	0	5/6	3/5	5/5
% Crop	0	0	0	3/6	2/3	3/3
% Wetland – forested	6/6	6/6	5/6	6/6	5/6	6/6
% Wetland – emergent	0	0	0	6/6	6/6	6/6
(C°) TMP	3/3	3/3	3/3	3/3	3/3	3/3
(mm) PCP	3/3	3/3	3/3	3/3	3/3	3/3
(C°) TMP.L1	3/3	3/3	3/3	2/3	2/2	2/2
(mm) PCP.L1	3/3	3/3	3/3	3/3	2/3	2/3
(Δ 30 yr normal) TMP.DFF	3/3	3/3	3/3	2/3	2/2	2/2
(Δ 30 yr normal) PCP.DFF	2/3	2/2	2/2	3/3	3/3	3/3
(Δ 30 yr normal) TMP.DFF.L1	3/3	3/3	3/3	2/3	2/2	2/2
(Δ 30 yr normal) PCP.DFF.L1	3/3	3/3	3/3	3/3	2/3	2/3
(scale from -10 to 10) PDSI	6/6	6/6	5/6	6/6	6/6	6/6
(scale from -10 to 10) PDSI.L1	6/6	6/6	6/6	6/6	6/6	6/6
(km^2) Population Density	6/6	3/6	6/6	6/6	5/6	6/6
(Avg. monthly trap night) Culex pipiens collections	6/6	6/6	6/6	---	---	---

Supplementary Table 2: Variable selection results from monthly West Nile virus detection and *Culex pipiens* collection training models. Units of each variable are listed in the variable list column. Abbreviations are as follows: TMP – temperature; PCP – precipitation; TMP.DFF – temperature difference from normal; PCP.DFF – precipitation difference from normal; PDSI – Palmer Drought Severity Index; L1 – one month lag; BRT – boosted regression tree; RF – random forest; GLM – generalized linear model; WNV – West Nile virus; CXP – *Cx. pipiens*. Numerators indicate the number of climate candidate models that kept the variable after a backward selection process. Denominators indicate the total number of climate candidate models tested. Denominators change for RF and GLM plots as variables were only assessed based on the final variables included in the simplified BRTs (i.e., the numerators).

Climate Variable Forms				Full model			Reduced model					
Current Month		Prior Month										
Temp	Precip	Temp	Precip	% CV deviance explained	% Residual Deviance explained	RMSE	N variables	Ranked Interactions	% CV deviance explained	% Residual Deviance explained	RMSE	Avg. performance rank
Average				71.1	91.8	7.93	Did not reduce					
Difference from normal		Average		66.7	92.5	7.36	**6**	**2**	**68.6**	**91.4**	**7.79**	**2.2**
Difference from normal	Average	Difference from normal	Average	66.6	92.0	7.11	17	14	66.7	91.7	7.25	3
Average	Difference from Normal	Average	Difference from normal	70.7	90.5	8.1	19	18	70.7	90.8	8.19	4
Average		Difference from normal		70.1	89.8	8.14	13	8	70.2	89.9	8.04	3.4
Difference from normal				66.0	93.0	7.43	12	7	67.5	92.2	7.52	2.4

Supplementary Table 3: Model performance of candidate boosted regression tree models of *Culex pipiens* monthly trap night collections at 87 sites in Connecticut, USA 2001 – 2020.

<table>
<tr><th colspan="4">Climate Variable Forms</th><th colspan="2" rowspan="2">Random Forest of simplified BRT</th><th colspan="4" rowspan="2">Further REDUCED random forest model</th></tr>
<tr><th colspan="2">Current Month</th><th colspan="2">Prior Month</th></tr>
<tr><th>Temp</th><th>Precip</th><th>Temp</th><th>Precip</th><th>% Variance explained</th><th>RMSE</th><th>N variables</th><th>% Variance explained</th><th>RMSE</th><th>Avg. performance rank</th></tr>
<tr><td colspan="4">Average</td><td>47.8</td><td>20.6</td><td colspan="3">Did not reduce</td><td></td></tr>
<tr><td colspan="2">Difference from normal</td><td colspan="2">Average</td><td>30.6</td><td>23.8</td><td>5</td><td>32.4</td><td>23.4</td><td>1.67</td></tr>
<tr><td>Difference from normal</td><td>Average</td><td>Difference from normal</td><td>Average</td><td>29.3</td><td>24.0</td><td>11</td><td>28.6</td><td>24.1</td><td>2</td></tr>
<tr><td>Average</td><td>Difference from Normal</td><td>Average</td><td>Difference from normal</td><td>47.6</td><td>20.6</td><td colspan="3">Did not reduce</td><td></td></tr>
<tr><td colspan="2">Average</td><td colspan="2">Difference from normal</td><td>47.0</td><td>20.8</td><td colspan="3">Did not reduce</td><td></td></tr>
<tr><td colspan="4">Difference from normal</td><td>30.2</td><td>23.8</td><td>11</td><td>30.8</td><td>23.7</td><td>2</td></tr>
</table>

Supplementary Table 4: Model performance of candidate random forest regression models of *Culex pipiens* monthly trap night collections at 87 sites in Connecticut, USA 2001 - 2020.

Climate Variable Forms				GLM of simplified BRT			Further REDUCED GLM			
Current Month		Prior Month								
Temp	Precip	Temp	Precip	N variables	% Residual Deviance explained	RMSE	N variables	% Residual Deviance explained	RMSE	Avg. performance rank
Average				254	68.1	1.13	226	68.1	1.13	3.67
Difference from normal		Average		22	27.6	1.03	20	27.6	1.04	3
Difference from normal	Average	Difference from normal	Average	154	47.4	1.42	131	47.4	1.41	4.3
Average	Difference from Normal	Average	Difference from normal	191	65.0	1.07	162	65.0	1.06	3.33
Average		Difference from normal		92	54.0	0.91	**84**	**54.0**	**0.91**	**2.33**
Difference from normal				79	39.6	1.44	74	39.6	1.44	4.33

Supplementary Table 5: Model performance of candidate general linear models of *Culex pipiens* monthly trap night collections at 87 sites in Connecticut, USA from 2001 – 2020.

Climate Variable Forms				Full model			Reduced model				
Current Month		Prior Month									
Temp	Precip	Temp	Precip	% Cross validated (CV) deviance explained	% Residual Deviance explained	Root Mean Square Error (RMSE)	N variables	% CV deviance explained (Rank)	% Residual Deviance explained	RMSE	Avg. performance rank
Average				49.9	68.9	0.001	13	50.7	69.7	0.0007	2.25
Difference from normal		Average		49.3	68.9	0.0002	**12**	**50.8**	**72.3**	**0.001**	**1.75**
Difference from normal	Average	Difference from normal	Average	43.0	65.8	0.002	10	46.3	69.2	0.003	3
Average	Difference from Normal	Average	Difference from normal	43.4	0.67	0.0006	10	45.9	68.9	0.002	3.25
Average		Difference from normal		43.4	67.3	0.0006	8	45.9	68.9	0.002	3
Difference from normal				43.0	67.8	0.002	10	45.1	68.5	0.003	4

Supplementary Table 6: Model performance of candidate boosted regression tree models of West Nile virus presence/absence in *Culex pipiens* mosquitoes captured at 87 sites in Connecticut, USA from 2001 – 2020

Climate Variable Forms				Random Forest of simplified BRT			Further REDUCED random forest model				
Current Month		Prior Month									
Temp	Precip	Temp	Precip	Balanced Accuracy	Sensitivity	Specificity	N variables	Balanced Accuracy	Sensitivity	Specificity	Avg. performance rank
Average				0.695	0.99	0.4	**12**	**0.71**	**0.99**	**0.44**	**1.25**
Difference from normal		Average		0.706	0.99	0.42	12	0.70	0.99	0.41	1.75
Difference from normal	Average	Difference from normal	Average	0.667	0.99	0.34	8	0.69	0.99	0.39	2.25
Average	Difference from Normal	Average	Difference from normal	0.695	0.99	0.4	8	0.69	0.99	0.40	2
Average		Difference from normal		0.658	0.99	0.324	8	0.69	0.99	0.38	2.5
Difference from normal				0.666	0.99	0.34	8	0.68	0.99	0.36	3

Supplementary Table 7: Model performance of candidate random forest classification models of West Nile virus presence/absence in *Culex pipiens* mosquitoes captured at 87 sites in Connecticut, USA from 2001 – 2020

Climate Variable Forms				GLM of simplified BRT			Further REDUCED GLM			
Current Month		Prior Month								
Temp	Precip	Temp	Precip	% Residual Deviance explained	RMSE	N variables	% Residual Deviance explained	RMSE	N variables	Avg. performance rank
Average				46.8	0.003	92	45.8	0.003	33	3
Difference from normal		Average		45.7	0.004	79	44.9	0.004	31	3.33
Difference from normal	Average	Differenc e from normal	Average	32.1	0.005	56	31.7	0.004	29	4
Average	Difference from Normal	Average	Difference from normal	43.2	0.003	37	**43.0**	**0.002**	**17**	**1.67**
Average		Difference from normal		35.0	0.006	56	34.6	0.006	26	3.67
Difference from normal				32.1	0.005	56	31.6	0.005	27	4.33

Supplementary Table 8: Model performance of candidate generalized linear models of West Nile virus presence/absence in *Culex pipiens* mosquitoes captured at 87 sites in Connecticut, USA from 2001 – 2020
